# Surgical and conservative treatment outcomes of medication-related osteonecrosis of the jaw located at tori: a retrospective study

**DOI:** 10.1007/s10006-024-01214-5

**Published:** 2024-02-29

**Authors:** Hameda Amin, Sanne Werner Moeller Andersen, Simon Storgård Jensen, Thomas Kofod

**Affiliations:** 1 Department of Odontology, Faculty of Health and Medical Sciences, University of Copenhagen, Copenhagen, Denmark; 2 Department of Oral & Maxillofacial Surgery, Copenhagen University Hospital, Copenhagen, Denmark; 3 Research Area Oral Surgery, Section for Oral Biology and Immunopathology, Department of Odontology, Faculty of Health and Medical Sciences, University of Copenhagen, Copenhagen, Denmark

**Keywords:** Bisphosphonate-associated osteonecrosis of the jaw, Exostoses, Surgery, Bone density conservation agents, Diphosphonates

## Abstract

**Purpose:**

Tori and exostoses are considered risk factors for the development of medication-related osteonecrosis of the jaw (MRONJ). The aims of this study were to present the prevalence of MRONJ located at tori in the Copenhagen ONJ Cohort, evaluate the surgical treatment of MRONJ located at tori and explore trauma to tori as an additional risk factor in patients on antiresorptive medication.

**Methods:**

Data from a consecutive series of 506 patients with MRONJ (Copenhagen ONJ Cohort) were reviewed for the presence of tori and MRONJ located at tori. Demographic and medical data were analyzed, and healing outcomes and pain after the prophylactic removal of tori, surgical treatment of MRONJ located at tori, and conservative treatment of MRONJ located at tori were evaluated and compared using Fisher’s exact test.

**Results:**

MRONJ located at tori was frequent and could be identified in 53% of the patients with tori, which accounts for a prevalence of 5.1% in the entire cohort. Of the 28 surgically treated patients, 27 (96.4%) healed uneventfully with no exposed bone after their first or second revision surgery. Fourteen (41.2%) patients with tori underwent therapeutic removal, eight (23.5%) underwent prophylactic removal, and six (17.6%) underwent both therapeutic and prophylactic removals. Two (33.3%) of the six conservatively treated patients healed spontaneously. Both treatment types resulted in a significant decrease in pain.

**Conclusion:**

Prophylactic and therapeutic surgical removal of tori are reliable treatments and should be considered if a patient’s general health allows surgery.

**Trial registration:**

The study was approved by the Regional Scientific Ethical Committee (H-6–2013-010) on November 20, 2013, and was retrospectively registered.

## Introduction

Medication-related osteonecrosis of the jaw (MRONJ) is a severe side effect of antiresorptive (AR) medication. AR medication includes bisphosphonates and denosumab and is used in low doses for patients with osteoporosis and as an adjuvant or in high doses for patients with various types of cancer [1–3]. MRONJ was initially described as bisphosphonate-induced osteonecrosis of the jaw by Marx [4] in 2003, which led to international recognition of the disease. Since then, an exponentially increasing number of publications have focused on MRONJ, especially on the incidence, risk factors, classification, and recommendations for treatment [5–10].

The risk of developing MRONJ is associated with the type of AR medication used and the cumulative exposure and potency of the medication [11]. The risk of developing MRONJ is higher for patients on high-dose AR medication (0.53–24%) than for patients on low-dose AR medication (0.02–0.05%) [12–17]. Various systemic and local risk factors have been linked to the development of MRONJ [18]. Systemic risk factors include concomitant treatment with immunosuppressants (corticosteroids and methotrexate) [10], smoking, poor oral hygiene and biofilm, chemotherapy and antiangiogenetic medication use, and comorbidities [19–25]. Among local risk factors, tooth extraction is the single most frequently reported predisposing factor for the development of MRONJ (62–82%) [5, 16, 26–28]. Pressure from ill-fitting dentures and local trauma during intubation and mastication as well as the presence of tori and exostoses are all considered local risk factors [27, 29–31].

Tori most frequently appear in the palate or mandible as torus palatinus or torus mandibularis, respectively, whereas exostoses most frequently appear buccally in the maxilla [32, 33]. Ueda et al. [34] reported the presence of tori in 18.6% of cancer patients on AR medication. Of these patients, 41.7% developed MRONJ located at their tori. Surgical treatment of MRONJ located at tori and/or the removal of tori in patients taking AR medication is currently controversial, and there are limited data on the treatment outcomes of MRONJ located at tori [35]. To the best of the authors’ knowledge, publications on patients taking AR medication who undergo surgical removal of tori are lacking.

The aims of this retrospective study were to (I) determine the prevalence of MRONJ located at tori in the Copenhagen ONJ Cohort, (II) evaluate the results of surgical treatment of MRONJ located at tori, (III) evaluate the results of the prophylactic removal of tori, and, finally, (IV) explore trauma to tori as a potential additional risk factor for patients with current or previous AR medication use in a consecutive series of patients referred to the Department of Oral & Maxillofacial Surgery, Copenhagen University Hospital for MRONJ treatment (The Copenhagen ONJ Cohort) [36].

## Materials and methods

The Copenhagen ONJ Cohort comprises 506 consecutive patients with MRONJ or patients taking AR medication who underwent surgical treatment. All patients were referred to the Department of Oral and Maxillofacial Surgery at Copenhagen University Hospital, Denmark, between 2005 and 2021. To evaluate conservative and surgical treatment of MRONJ located at tori including prophylactic removal of tori, the inclusion criteria were as follows: (1) the presence of tori with or without MRONJ, (2) current or previous treatment with AR medication and (3) no history of radiation to the jaws. The exclusion criterion was patients with no tori present. Furthermore, to determine the prevalence of MRONJ located at tori the inclusion criteria were as follows: (1) patients with MRONJ, (2) current or previous treatment with AR medication and (3) no history of radiation to the jaws. There were no exclusion criteria for this purpose.

Demographic data, including medication use, main diagnosis, clinical and radiographic examination results, and MRONJ staging were collected. Pre- and postoperative pain (Visual Analogue Scale, VAS) was recorded for both surgically treated and conservatively treated patients. The number of patients who stopped AR medication use prior to treatment was also recorded. The prevalence of MRONJ located at tori in the cohort was calculated as well as the frequency of MRONJ located at tori among patients with tori present. Patients signed informed consent forms, and conservative and surgical treatment was performed according to the Danish Standard Operation Procedure for MRONJ (accessible from the Department of Oral & Maxillofacial Surgery, Copenhagen University Hospital). Conservative treatment was chosen in cases where the general health of the patient did not allow surgical treatment or if sequestra were present and removed at the time of the initial examination and healing could be expected. Surgical treatment was chosen in most cases where healing was not expected. In cases where patients had MRONJ that was not located at the tori, the tori were removed prophylactically as part of the surgical treatment for MRONJ. The study was approved by The Danish Data Protection Agency (P-2022–856) and the Regional Scientific Ethical Committee (H-6–2013-010).

### Conservative treatment

For patients undergoing conservative treatment, optimal oral hygiene was obtained, chlorhexidine mouth rinse was prescribed, and systemic antibiotics were administered in cases of infection. Follow-up examinations were performed at 1, 3, 6, 9, and 12 months and yearly thereafter.

### Surgical treatment

Perioperatively, 500 mg amoxicillin/125 mg clavulanic acid tablets (three times daily) were administered prophylactically starting one day prior to surgery and continued for 10 days postoperatively. For patients treated with general anesthesia, 1500 mg cefuroxime was administered perioperatively. Patients with penicillin allergy were administered 300 mg clindamycin three times daily.

Surgical procedures were performed under sterile conditions with the patient either under general anesthesia and local anesthesia with 0.5% Marcaine Adrenaline as a supplement or under local anesthesia with lidocaine epinephrine (10 mg/ml, 5 ug/ml). Chlorhexidine 0.12% was used to rinse the oral cavity prior to the surgical procedure.

**Fig. 1:**
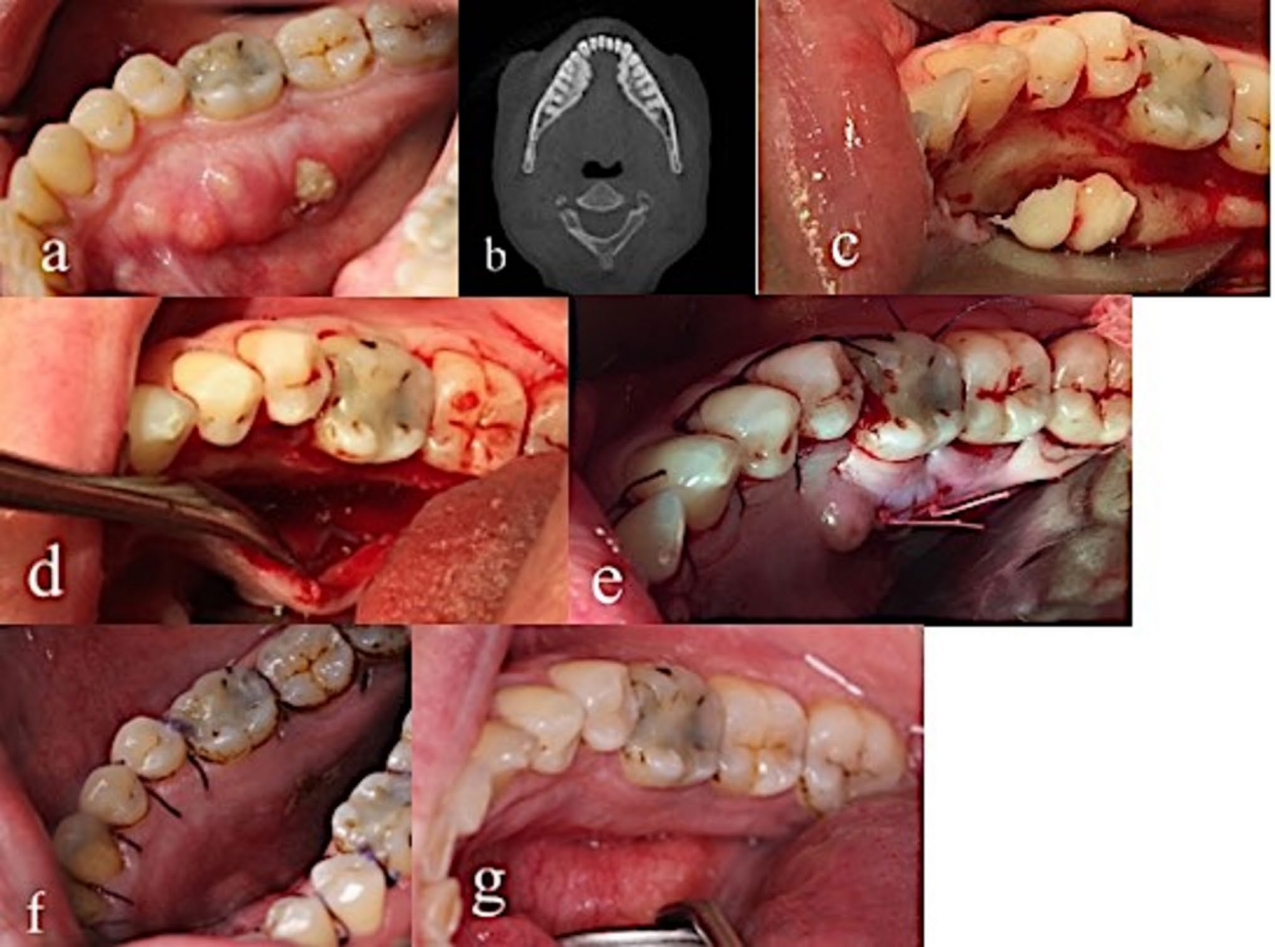
A 50-year-old woman with breast cancer and metastases to the femur was treated with denosumab 120 mg/month for 11 months. **a** A 6 × 6-mm MRONJ lesion located on the torus on the right side of the mandible. VAS score = 9. **b** Cone beam computed tomography (CBCT) showing radiolucency in the torus on the right side of the mandible compatible with osteonecrosis. **c** Tori and necrotic bone were removed. **d** Sharp edges were smoothed until fresh bleeding from the remaining bone was observed. **e** Primary closure. **f** Two weeks after surgery. **g** 17 months after surgery

A sulcular incision was made in the area of the exposed bone and torus. A mucoperiosteal flap was raised from the underlying bone, and the torus was removed together with the necrotic bone using a fissure bur with copious saline irrigation. Sharp bone edges were smoothed with a round bur until fresh bleeding from the remaining bone was observed. Finally, the area was rinsed with sterile saline. Primary tension-free wound closure was ensured by mobilizing the mucoperiosteal flap. In patients with larger defects, the mylohyoid muscle was utilized as a pedicled muscle flap. The flap was sutured with resorbable sutures (Ethicon Vicryl Suture 4–0, Johnson & Johnson). An example of the surgical procedure in a 50-year-old patient is shown in Fig. 1.

Postoperatively, the patients rinsed with chlorhexidine 0.12% twice daily until suture removal approximately 14 days after the surgery. Patients were seen at follow-up visits to evaluate and record the healing outcome after 1, 3, 6, 9, and 12 months and yearly thereafter.

### Statistics

SPSS (IBM SPSS Statistics for Windows, Version 25.0 Armonk, NY, IBM Corp.) was used to perform statistical analysis of the data. Statistically significant differences in healing outcomes between conservatively treated and surgically treated patients were compared using Fisher’s exact test. Furthermore, the Wilcoxon signed-rank test was used to compare preoperative and postoperative VAS scores. The results were considered statistically significant if the *P* value was < 0.05.

## Results

The prevalence of MRONJ located at tori was 5.1% as 26 patients with MRONJ located at tori were identified out of 506 included patients in the cohort. Furthermore, these 26 patients with MRONJ located at the tori accounts for 53% of the 49 patients with the presence of tori. Regarding healing outcomes of conservatively and surgically treated patients with tori, 49 patients with tori or exostoses could be identified in the cohort, of whom 34 were either surgically or conservatively treated. Thirty-two patients were diagnosed with MRONJ stage 1, 2, or 3, and two patients did not have MRONJ; instead, their tori were removed due to discomfort. A total of 28 patients were surgically treated, with tori removed prophylactically, therapeutically, or both prophylactically and therapeutically, and six patients were treated conservatively.

### Baseline characteristics

The demographic characteristics of the 34 conservatively or surgically treated patients are shown in Table 1. The study included 19 (55.9%) females and 15 (44.1%) males. A total of 14 (41.2%) patients were diagnosed with cancer, 16 (47.0%) were diagnosed with osteoporosis, and four (11.8%) were diagnosed with osteoporosis and cancer. All cancer patients received high-dose AR medications. In total, 19 (55.9%) patients received only bisphosphonates, 11 (32.3%) received only denosumab, and four (11.8%) received both. The mean duration of bisphosphonate and denosumab treatment was 43.6 months (SD ± 36.6, range 1–120) and 28.2 months (SD ± 23.6, range 2–68), respectively. The mean duration (months) of low-dose AR medication was 55.3 ± 37.9 (3–120) for bisphosphonate-treated patients and 56 ± 25.9 (24–68) for denosumab-treated patients. Furthermore, the mean duration of high-dose AR medication was 18.8 ± 17.0 months (1–55) for bisphosphonate-treated patients and 16.6 ± 11.2 months (2–36) for denosumab-treated patients.

**Table 1:** Demographics of the 34 treated patients divided by AR medication dosage

	Low dose Patients with osteoporosis	High dose Patients with cancer	Total
Age, years—mean ± SD (range)	73.00 ± 9.1 (54–88)	66.5 ± 8.8 (51–78)	69.5 ± 9.5 (51–88)
Sex, *n* (%)			
1. Male 2. Female	4 (11.8) 12 (35.3)	11 (32.3) 7 (20.6)	15 (44.1) 19 (55.9)
General disease, *n* (%)			
1. Breast cancer		5 (14.7)	5 (14.7)
2. Prostate cancer		3 (8.8)	3 (8.8)
3. Osteoporosis	16 (47.0)		16 (47.0)
4. Multiple myeloma		3 (8.8)	3 (8.8)
5. Lung cancer		1 (2.9)	1 (2.9)
6. Kidney cancer		1 (2.9)	1 (2.9)
7. Cardia cancer		1 (2.9)	1 (2.9)
8. Osteoporosis + cancer		4 (11.8)	4 (11.8)
Antiresorptive treatment, *n* (%)			
1. Bisphosphonate only			
- Oral - IV	12^a^ (35.3) 1 (2.9)	1 (2.9) 5^b^ (14.7)	13 (38.2) 6 (17.6)
2. Denosumab only			
- Sc	3^c^ (8.8)	8^d^ (23.5)	11 (32.3)
3. Bisphosphonate + denosumab			
- Oral - IV - Sc	0 0 0	0 0 4 (11.8)	0 0 4^e^ (11.8)
Duration of AR treatment, month—mean ± SD (range)			
1. Bisphosphonate 2. Denosumab	55.3 ± 37.9 (3–120) 56 ± 25.9 (24–68)	18.8 ± 17.0 (1–55) 16.6 ± 11.2 (2–36)	43.6 ± 36.6 (1–120) 28.2 ± 23.6 (2–68)
AR discontinued preoperatively, *n* (%)	8 (23.5)	15 (44.1)	23 (67.6)
Comorbidity, *n* (%)			
1. Steroid treatment 2. Chemotherapy 3. Tobacco use, current or previous	2 (5.8) 0 (0.0) 11 (32.3)	10 (29.4) 13 (38.2) 8 (23.5)	12 (35.3) 13 (38.2) 19 (55.9)
Trauma mechanism, *n* (%)			
1. Tooth extraction	2 (5.8)	5 (14.7)	7 (20.6)
2. Prosthesis	2 (5.8)	1 (2.9)	3 (8.8)
3. Intubation	0 (0.0)	1 (2.9)	1 (2.9)
4. Food	0 (0.0)	1 (2.9)	1 (2.9)
5. Toothbrushing	0 (0.0)	1 (2.9)	1 (2.9)
6. Unknown	10 (29.4)	9 (26.5)	19 (51.3)
7. Not relevant	2 (5.8)		2 (5.8)
Stage, *n* (%)			
1. Stage 0 2. Stage 1 3. Stage 2 4. Stage 3 5. No MRONJ	0 (0.0) 7 (20.6) 4 (11.8) 3 (8.8) 2 (5.8)	0 (0.0) 4 (11.8) 10 (29.4) 4 (11.8) 0 (0.0)	0 (0.0) 11 (32.3) 14 (41.2) 7 (20.6) 2 (5.8)
Location of MRONJ, *n* (%)			
1. At the site of the torus 2. Sites other than that of the torus 3. Both sides 4. None	13 (38.2) 2 (5.8) 0.0 2 (5.8)	11 (32.3) 4 (11.8) 2 (5.8) 0	24 (70.5) 6 (17.6) 2 (5.8) 2 (5.8)
Follow-up, months—mean ± SD (range)	16.4 ± 16.0 (1–56)	18.5 ± 17.9 (3–79)	17.5 ± 16.8 (1–79)
VAS score—mean ± SD (range)			
1. Preoperatively 2. Postoperatively	1.8 ± 2.5 (0–7) .0 ± .0 (0–0)	3.6 ± 3.5 (0–10) .0 ± .0 (0–0)	2.9 ± 3.35 (0–10) .0 ± .0 (0–0)
Death, *n* (%)	2 (5.8)	10 (29.4)	12 (35.3)
Treatment, *n* (%)			
1. Surgical 2. Nonsurgical	14 (41.2) 2 (5.8)	14 (41.2) 4 (11.8)	28 (82.3) 6 (17.6)
Healing, *n* (%)			
- Yes - No	15 (44.1) 1 (2.9)	14 (41.2) 4 (11.8)	29 (85.3) 5 (14.7)
Indication for removal of tori, *n* (%)			
1. Prophylactic 2. Therapeutic 3. Both	4 (11.8) 7 (20.6) 3 (8.8)	4 (11.8) 7 (20.6) 3 (8.8)	8 (23.5) 14 (41.2) 6 (17.6)
Location of treated tori			
1. Buccal exostoses 2. Mandibular tori 3. Buccal exostoses + mandibular tori	(0.0) 15 (44.1) 1 (2.9)	1 (2.9) 16 (47.0) 1 (2.9)	1 (2.9) 31 (91.2) 2 (5.8)

*IV* intravenous administration, *SC* subcutaneous injection, *MRONJ* medication-related osteonecrosis of the jaw

^a^Three patients also received methotrexate

^b^One patient also received a low-dose bisphosphonate, and one patient received two different high-dose bisphosphonates

^c^One patient also received methotrexate

^d^One patient received axitinib, sunitinib, cabozantinib, and nivolumab in addition to denosumab

^e^All patients in this group had received both bisphosphonates and denosumab: one patient received a low-dose bisphosphonate intravenously and denosumab, two patients received a low-dose bisphosphonate (tablet) combined with denosumab, and one patient received a high-dose bisphosphonate combined with denosumab

A total of 11 (32.3%) patients were diagnosed with MRONJ stage 1, 14 (41.2%) were diagnosed with stage 2, and seven (20.6%) were diagnosed with stage 3. The MRONJ lesion was located at tori in 24 (70.5%) patients, while the lesion was localized at a site other than that of the tori in six patients (17.6%); hence, some patients underwent prophylactic removal of the tori as part of their surgical treatment for MRONJ. Two patients (5.8%) had bilateral MRONJ at the site of the tori as well as at other sites. Tori were removed therapeutically in 14 (41.2%) patients and prophylactically in eight (23.5%) patients. In six (17.6%) patients, tori were removed therapeutically on one side and prophylactically on the other.

### Anatomic location prone to trauma

Mandibular tori were diagnosed in 31 (91.2%) of the 34 treated patients; in two (5.8%) patients, buccal as well as lingual exostoses were present, and one (2.9%) patient had buccal exostoses only. Of the 34 patients, 26 (76.5%) had MRONJ located at the tori. Only 13 (38.2%) patients had a known trauma history prior to the occurrence of the exposed bone. Seven (20.6%) patients had undergone a tooth extraction, three (8.8%) had pressure sores from an ill-fitted removable dental prosthesis, one (2.9%) suffered trauma during intubation, one (2.9%) suffered trauma from hard food items, and one (2.9%) developed an ulcer from tooth-brushing. Of the 13 patients with a known trauma history, seven had MRONJ located at the tori. Hence, of the 26 patients with MRONJ located at the tori, 27% had suffered a known trauma prior to the occurrence of the MRONJ lesion; therefore, the other six cases of trauma led to MRONJ at other sites.

### Healing outcomes

In total, 29 (85.3%) of the 34 patients healed. Of the 28 surgically treated patients, 27 (96.4%) healed; in three of these patients, a second revision surgery was indicated. Two of the three initially unhealed patients had a local dehiscence after the primary surgery, and one surgically treated patient (3.6%) remained unhealed after a second revision surgery. All patients with the need for secondary revision surgery were diagnosed with MRONJ located at the tori. Thus, all patients who underwent the prophylactic removal of tori healed uneventfully with no exposed bone after the first surgery.

Conservative treatment was chosen in six (17.6%) patients diagnosed with MRONJ located at the tori. Two (33.3%) of these patients healed spontaneously, while four (66.6%) remained unhealed. Hence, surgical treatment of tori led to better healing outcomes than conservative treatment (*P* < 0.05). Three unhealed and conservatively treated patients died after only three months of observation.

The mean follow-up was 17.5 months (SD ± 16.8, range 1–79 months), with 21 (61.7%) patients being followed for one year or more. Surgical as well as conservative treatment significantly reduced the VAS pain score from 2.96 before treatment to 0.0 after treatment (*P* < 0.05).

AR treatment was discontinued in eight (23.5%) patients treated for osteoporosis and 15 (44.1%) patients treated for cancer prior to treatment, with an overall discontinuation rate of 67.6%.

## Discussion

In the present study, a prevalence of 5.1% was identified for MRONJ located at tori in the Copenhagen ONJ Cohort. The success rate after surgical treatment of MRONJ located at tori was 96.4%, while only two (33.3%) conservatively treated patients healed spontaneously. Furthermore, in this study, a 100% success rate after the prophylactic removal of tori was reported.

In the present study, 26 (76.5%) patients had MRONJ located at tori, accounting for 53.0% of the 49 patients with tori in the entire Copenhagen ONJ Cohort. Similarly, Ueda et al. [34] found that 41.7% of cancer patients on AR medication developed MRONJ at the site of their tori.

The oral mucosa covering tori and exostoses is thin and less vascularized compared to mucosa elsewhere in the oral cavity [37]. AR treatment has been documented as compromising angiogenesis [38], and being predominantly cortical bone growths, tori are expected to have a reduced healing capacity, explaining the high prevalence of MRONJ at these anatomical locations. Furthermore, tori are prominent structures and hence increasingly susceptible to trauma. In the present study, 18 (69.2%) of the 26 patients with MRONJ located at their tori could not recall if they had endured any kind of trauma, while seven (27%) could report a specific trauma incident as the presumed cause of their exposed tori. Trauma may thus be an additional risk factor for patients with tori. Therefore, dentists should be aware of the risks in these patients when tori are present, and furthermore, dentists must be cautious when treating them. This means caution must be taken when taking impressions for dentures or other dental prostheses for these patients. Dentists should also prevent the use of ill-fitting dentures and try to avoid trauma from suction devices during dental treatment. Other medical staff should also be cautious when treating these patients, for instance, during intubation.

Nicolatou-Galitis et al. [39] found that patients taking high-dose AR medication in general had a higher risk of developing MRONJ than patients taking low-dose AR medication. However, the dose of AR medication had no impact on the risk of developing MRONJ in the present data set, where 50% of the cases of MRONJ located at tori were observed in patients on low-dose AR medications. This may again be because the mucosa on exostoses and tori is thin, less vascularized, and susceptible to trauma, which makes it prone to developing MRONJ in general; however, most likely, this finding may be connected to the size of the group on low-dose AR medication, which was much larger than that of the group on high-dose AR medication.

To the best of the authors’ knowledge, there are no publications evaluating the treatment strategy of tori in patients on AR medication. The success rate of surgical treatment was 89.3% (25 of 28 patients), which increased to 96.4% (27 of 28) after three patients underwent a second surgery. Only two (33.3%) patients in the conservatively treated group healed, and a statistically significant difference in the healing outcome between the two groups was found (*P* < 0.05). This indicates that surgical treatment can be a successful treatment strategy and furthermore, that conservatively treated patients have a low healing rate compared to surgically treated patients. However, the low healing rate among the conservatively treated patients may also be linked to their general health since a compromised general health status was the reason for choosing a conservative approach in 17.6% of the cases. Regarding conservative treatment of patients with MRONJ located at the tori, the survival rate after three months is 50%. Conservative treatment was chosen in some cases due to the compromised general health of the patient, which may be reflected in the high mortality rate.

Furthermore, 20 (71.4%) of the 28 patients undergoing surgery had their tori removed therapeutically, while eight (28.6%) had their tori removed prophylactically. The three patients who did not heal after the first surgery were all therapeutically treated patients, and all prophylactically treated patients healed uneventfully after the first surgery. This finding may indicate that the healing potential is better in cases where tori are removed prophylactically; therefore, prophylactic treatment could be considered for the removal of large tori in patients with current or previous AR medication use.

In the present study, 67.6% of the patients stopped AR medication use prior to treatment. Among these patients, 15 (44.1%) were taking high-dose AR medication. Drug discontinuation has been hypothesized to lower the risk of developing MRONJ after dentoalveolar surgery and promote complete healing thereafter. However, in a randomized clinical study, Ottesen et al. [40] investigated the efficacy of a drug holiday on the development of MRONJ in cancer patients taking high-dose AR medication prior to tooth extraction. It was found that drug holidays for patients on high-dose AR medication did not prevent the development of MRONJ after tooth extraction, and furthermore, the patient-reported health state declined for patients on drug holidays compared to patients who continued treatment. Hence, drug holidays are not a prerequisite for complete healing, and this should be taken into consideration when treating this group of patients, especially patients on high-dose AR medication.

The present retrospective study has several limitations. The Copenhagen ONJ cohort is a selected group of patients on AR medication, and the prevalence of tori in this cohort is therefore not necessarily representative of all patients on AR medication. No randomization of conservative or surgical treatment was performed, and the group of patients receiving conservative treatment was small and not comparable to the group receiving surgical treatment. Therefore, the comparison of healing outcomes should be performed with great caution. The cohort was relatively small and heterogeneous, including patients taking low-dose and high-dose AR medications for different durations, patients with and without comorbidities and patients undergoing therapeutic, prophylactic, or conservative treatment of their tori, making firm conclusions difficult. However, to the best of the authors’ knowledge, this study included the largest group of patients on AR medication, systematically evaluating different tori treatment approaches and healing outcomes.

To support the present findings, further prospective studies with larger sample sizes are needed.

## Conclusion

MRONJ frequently develops on mandibular tori or buccal exostoses in patients on high-dose and low-dose AR medications. A known type of trauma could be identified in one-third of the patients. The prophylactic and therapeutic removal of tori was predictably followed by complete healing, whereas conservative treatment rarely resulted in healing. Future studies should focus on indications for the prophylactical removal of tori in patients taking AR medication.

## Data Availability

Not available.

## Code availability

Not available.

## References

[1] Coleman R, Body JJ, Aapro M, Hadji P, Herrstedt J (2014) Bone health in cancer patients: ESMO clinical practice guidelines. Ann Oncol 25:iii124–iii137. 10.1093/annonc/mdu103 24782453 10.1093/annonc/mdu103

[2] Burkiewicz JS, Scarpace SL, Bruce SP (2009) Denosumab in osteoporosis and oncology. Ann Pharmacother 43:1445–1455. 10.1345/aph.1M102 19622756 10.1345/aph.1M102

[3] Drake MT, Clarke BL, Khosla S (2008) Bisphosphonates: mechanism of action and role in clinical practice. Mayo Clin Proc 83:1032–1045. 10.4065/83.9.1032 18775204 10.4065/83.9.1032 PMC2667901

[4] Marx RE (2003) Pamidronate (Aredia) and zoledronate (Zometa) induced avascular necrosis of the jaws: a growing epidemic. J Oral Maxillofac Surg 61:1115–1117. 10.1016/s0278-2391(03)00720-1 12966493 10.1016/s0278-2391(03)00720-1

[5] Ruggiero SL, Mehrotra B, Rosenberg TJ, Engroff SL (2004) Osteonecrosis of the jaws associated with the use of bisphosphonates: a review of 63 cases. J Oral Maxillofac Surg 62:527–534. 10.1016/j.joms.2004.02.004 15122554 10.1016/j.joms.2004.02.004

[6] Ruggiero S, Gralow J, Marx RE, Hoff AO, Schubert MM, Huryn JM, Toth B, Damato K, Valero V (2006) Practical guidelines for the prevention, diagnosis, and treatment of osteonecrosis of the jaw in patients with cancer. J Oncol Pract 2:7–14. 10.1200/jop.2006.2.1.7 20871729 10.1200/jop.2006.2.1.7 PMC2794643

[7] Ruggiero SL, Dodson TB, Assael LA, Landesberg R, Marx RE, Mehrotra B (2009) American association of oral and maxillofacial surgeons position paper on bisphosphonate-related osteonecrosis of the jaws - 2009 update. Aust Endod J 35:119–130. 10.1111/j.1747-4477.2009.00213.x 19961450 10.1111/j.1747-4477.2009.00213.x

[8] Schiodt M, Reibel J, Oturai P, Kofod T (2014) Comparison of nonexposed and exposed bisphosphonate-induced osteonecrosis of the jaws: a retrospective analysis from the Copenhagen cohort and a proposal for an updated classification system. Oral Surg Oral Med Oral Pathol Oral Radiol 117:204–213. 10.1016/j.oooo.2013.10.010 24332520 10.1016/j.oooo.2013.10.010

[9] Otto S, Pautke C, Van den Wyngaert T, Niepel D, Schiødt M (2018) Medication-related osteonecrosis of the jaw: prevention, diagnosis and management in patients with cancer and bone metastases. Cancer Treat Rev 69:177–187. 10.1016/j.ctrv.2018.06.007 30055439 10.1016/j.ctrv.2018.06.007

[10] Ruggiero SL, Dodson TB, Aghaloo T, Carlson ER, Ward BB, Kademani D (2022) American association of oral and maxillofacial surgeons’ position paper on medication-related osteonecrosis of the jaws-2022 update. J Oral Maxillofac Surg 80:920–943. 10.1016/j.joms.2022.02.008 35300956 10.1016/j.joms.2022.02.008

[11] Henry DH, Costa L, Goldwasser F et al (2011) Randomized, double-blind study of denosumab versus zoledronic acid in the treatment of bone metastases in patients with advanced cancer (excluding breast and prostate cancer) or multiple myeloma. J Clin Oncol 29:1125–1132. 10.1200/jco.2010.31.3304 21343556 10.1200/jco.2010.31.3304

[12] Raje N, Terpos E, Willenbacher W et al (2018) Denosumab versus zoledronic acid in bone disease treatment of newly diagnosed multiple myeloma: an international, double-blind, double-dummy, randomised, controlled, phase 3 study. Lancet Oncol 19:370–381. 10.1016/s1470-2045(18)30072-x 29429912 10.1016/s1470-2045(18)30072-x

[13] Valachis A, Polyzos NP, Coleman RE, Gnant M, Eidtmann H, Brufsky AM, Aft R, Tevaarwerk AJ, Swenson K, Lind P, Mauri D (2013) Adjuvant therapy with zoledronic acid in patients with breast cancer: a systematic review and meta-analysis. Oncologist 18:353–361. 10.1634/theoncologist.2012-0261 23404816 10.1634/theoncologist.2012-0261 PMC3639520

[14] Coleman R, Finkelstein DM, Barrios C et al (2020) Adjuvant denosumab in early breast cancer (D-CARE): an international, multicentre, randomised, controlled, phase 3 trial. Lancet Oncol 21:60–72. 10.1016/s1470-2045(19)30687-4 31806543 10.1016/s1470-2045(19)30687-4

[15] Saag KG, Petersen J, Brandi ML, Karaplis AC, Lorentzon M, Thomas T, Maddox J, Fan M, Meisner PD, Grauer A (2017) Romosozumab or alendronate for fracture prevention in women with osteoporosis. N Engl J Med 377:1417–1427. 10.1056/NEJMoa1708322 28892457 10.1056/NEJMoa1708322

[16] Hallmer F, Andersson G, Götrick B, Warfvinge G, Anderud J, Bjørnland T (2018) Prevalence, initiating factor, and treatment outcome of medication-related osteonecrosis of the jaw-a 4-year prospective study. Oral Surg Oral Med Oral Pathol Oral Radiol 126:477–485. 10.1016/j.oooo.2018.08.015 30249535 10.1016/j.oooo.2018.08.015

[17] Badros A, Weikel D, Salama A, Goloubeva O, Schneider A, Rapoport A, Fenton R, Gahres N, Sausville E, Ord R, Meiller T (2006) Osteonecrosis of the jaw in multiple myeloma patients: clinical features and risk factors. J Clin Oncol 24:945–952. 10.1200/jco.2005.04.2465 16484704 10.1200/jco.2005.04.2465

[18] Khan AA, Morrison A, Hanley DA et al (2015) Diagnosis and management of osteonecrosis of the jaw: a systematic review and international consensus. J Bone Miner Res 30:3–23. 10.1002/jbmr.2405 25414052 10.1002/jbmr.2405

[19] Viviano M, Rossi M, Cocca S (2017) A rare case of osteonecrosis of the jaw related to imatinib. J Korean Assoc Oral Maxillofac Surg 43:120–124. 10.5125/jkaoms.2017.43.2.120 28462197 10.5125/jkaoms.2017.43.2.120 PMC5410424

[20] Dişel U, Beşen AA, Özyılkan Ö, Er E, Canpolat T (2012) A case report of bevacizumab-related osteonecrosis of the jaw: old problem, new culprit. Oral Oncol 48:e2–e3. 10.1016/j.oraloncology.2011.07.030 21865077 10.1016/j.oraloncology.2011.07.030

[21] Allegra A, Oteri G, Alonci A, Bacci F, Penna G, Minardi V, Maisano V, Musolino C (2014) Association of osteonecrosis of the jaws and POEMS syndrome in a patient assuming rituximab. J Craniomaxillofac Surg 42:279–282. 10.1016/j.jcms.2013.05.014 23800756 10.1016/j.jcms.2013.05.014

[22] Vandone AM, Donadio M, Mozzati M, Ardine M, Polimeni MA, Beatrice S, Ciuffreda L, Scoletta M (2012) Impact of dental care in the prevention of bisphosphonate-associated osteonecrosis of the jaw: a single-center clinical experience. Ann Oncol 23:193–200. 10.1093/annonc/mdr039 21427065 10.1093/annonc/mdr039

[23] Sedghizadeh PP, Kumar SK, Gorur A, Schaudinn C, Shuler CF, Costerton JW (2009) Microbial biofilms in osteomyelitis of the jaw and osteonecrosis of the jaw secondary to bisphosphonate therapy. J Am Dent Assoc 140:1259–1265. 10.14219/jada.archive.2009.0049 19797556 10.14219/jada.archive.2009.0049

[24] Hayano H, Kuroshima S, Sasaki M, Tamaki S, Inoue M, Ishisaki A, Sawase T (2020) Distinct immunopathology in the early stages between different antiresorptives-related osteonecrosis of the jaw-like lesions in mice. Bone 135:115308. 10.1016/j.bone.2020.115308 32142911 10.1016/j.bone.2020.115308

[25] Aghaloo TL, Tetradis S (2017) Osteonecrosis of the jaw in the absence of antiresorptive or antiangiogenic exposure: a series of 6 cases. J Oral Maxillofac Surg 75:129–142. 10.1016/j.joms.2016.07.019 27569557 10.1016/j.joms.2016.07.019 PMC5527276

[26] Yazdi PM, Schiodt M (2015) Dentoalveolar trauma and minor trauma as precipitating factors for medication-related osteonecrosis of the jaw (ONJ): a retrospective study of 149 consecutive patients from the Copenhagen ONJ cohort. Oral Surg Oral Med Oral Pathol Oral Radiol 119:416–422. 10.1016/j.oooo.2014.12.024 25697927 10.1016/j.oooo.2014.12.024

[27] Hoff AO, Toth BB, Altundag K et al (2008) Frequency and risk factors associated with osteonecrosis of the jaw in cancer patients treated with intravenous bisphosphonates. J Bone Miner Res 23:826–836. 10.1359/jbmr.080205 18558816 10.1359/jbmr.080205 PMC2677083

[28] Saad F, Brown JE, Van Poznak C et al (2012) Incidence, risk factors, and outcomes of osteonecrosis of the jaw: integrated analysis from three blinded active-controlled phase III trials in cancer patients with bone metastases. Ann Oncol 23:1341–1347. 10.1093/annonc/mdr435 21986094 10.1093/annonc/mdr435

[29] Wick A, Bankosegger P, Otto S, Hohlweg-Majert B, Steiner T, Probst F, Ristow O, Pautke C (2022) Risk factors associated with onset of medication-related osteonecrosis of the jaw in patients treated with denosumab. Clin Oral Investig 26:2839–2852. 10.1007/s00784-021-04261-4 34812959 10.1007/s00784-021-04261-4 PMC8898220

[30] Marx RE, Sawatari Y, Fortin M, Broumand V (2005) Bisphosphonate-induced exposed bone (osteonecrosis/osteopetrosis) of the jaws: risk factors, recognition, prevention, and treatment. J Oral Maxillofac Surg 63:1567–1575. 10.1016/j.joms.2005.07.010 16243172 10.1016/j.joms.2005.07.010

[31] O’Ryan FS, Lo JC (2012) Bisphosphonate-related osteonecrosis of the jaw in patients with oral bisphosphonate exposure: clinical course and outcomes. J Oral Maxillofac Surg 70:1844–1853. 10.1016/j.joms.2011.08.033 22595135 10.1016/j.joms.2011.08.033

[32] Sawair FA, Shayyab MH, Al-Rababah MA, Saku T (2009) Prevalence and clinical characteristics of tori and jaw exostoses in a teaching hospital in Jordan. Saudi Med J 30:1557–1562 19936420

[33] AlZarea BK (2016) Prevalence and pattern of torus palatinus and torus mandibularis among edentulous patients of Saudi Arabia. Clin Interv Aging 11:209–213. 10.2147/cia.s100282 26966357 10.2147/cia.s100282 PMC4771409

[34] Ueda N, Aoki K, Shimotsuji H, Nakashima C, Kawakami M, Imai Y, Kirita T (2021) Oral risk factors associated with medication-related osteonecrosis of the jaw in patients with cancer. J Bone Miner Metab 39:623–630. 10.1007/s00774-020-01195-x 33420576 10.1007/s00774-020-01195-x

[35] Goldman ML, Denduluri N, Berman AW, Sausville R, Guadagnini JP, Kleiner DE, Brahim JS, Swain SM (2006) A novel case of bisphosphonate-related osteonecrosis of the torus palatinus in a patient with metastatic breast cancer. Oncology 71:306–308. 10.1159/000106451 17657174 10.1159/000106451

[36] Corraini P, Heide-Jørgensen U, Schiødt M, Nørholt SE, Acquavella J, Sørensen HT, Ehrenstein V (2017) Osteonecrosis of the jaw and survival of patients with cancer: a nationwide cohort study in Denmark. Cancer Med 6:2271–2277. 10.1002/cam4.1173 28941210 10.1002/cam4.1173 PMC5633555

[37] García-García AS, Martínez-González JM, Gómez-Font R, Soto-Rivadeneira A, Oviedo-Roldán L (2010) Current status of the torus palatinus and torus mandibularis. Med Oral Patol Oral Cir Bucal 15:e353–e360. 10.4317/medoral.15.e353 19767716 10.4317/medoral.15.e353

[38] Allegra A, Innao V, Pulvirenti N, Musolino C (2019) Antiresorptive agents and anti-angiogenesis drugs in the development of osteonecrosis of the jaw. Tohoku J Exp Med 248:27–29. 10.1620/tjem.248.27 31080196 10.1620/tjem.248.27

[39] Nicolatou-Galitis O, Schiødt M, Mendes RA, Ripamonti C, Hope S, Drudge-Coates L, Niepel D, Van Den Wyngaert T (2019) Medication-related osteonecrosis of the jaw: definition and best practice for prevention, diagnosis, and treatment. Oral Surg Oral Med Oral Pathol Oral Radiol 127:117–135. 10.1016/j.oooo.2018.09.008 30393090 10.1016/j.oooo.2018.09.008

[40] Ottesen C, Schiodt M, Jensen SS, Kofod T, Gotfredsen K (2022) Tooth extractions in patients with cancer receiving high-dose antiresorptive medication: a randomized clinical feasibility trial of drug holiday versus drug continuation. Oral Surg Oral Med Oral Pathol Oral Radiol 133:165–173. 10.1016/j.oooo.2021.06.003 34275774 10.1016/j.oooo.2021.06.003

